# Drug-Induced Serious Cutaneous Reactions in Hospitalized Patients: A Cross-Sectional Study

**DOI:** 10.3390/jcm14030857

**Published:** 2025-01-28

**Authors:** Warisara Jiamsathit, Kansuda Bunarong, Sonthiya Papenkort, Anthony R. Cox, Narumol Jarernsiripornkul

**Affiliations:** 1 Sirindhorn College of Public Health Khon Kaen, Faculty of Public Health and Allied Health Sciences, Praboromarajchanok Institute, Khon Kaen 40000, Thailand; warisara@scphkk.ac.th; 2 Division of Clinical Pharmacy, Faculty of Pharmaceutical Sciences, Khon Kaen University, Khon Kaen 40002, Thailand; kansudab.bb@gmail.com (K.B.); sonthiya.kort@gmail.com (S.P.); 3 Department of Pharmacy, School of Health Sciences, College of Medicine and Health, University of Birmingham, Birmingham B15 2TT, UK; a.r.cox@bham.ac.uk

**Keywords:** adverse drug reactions, severe cutaneous adverse reaction, serious skin reaction

## Abstract

**Background:** Serious adverse drug reactions (ADRs) can lead to hospital admission and can be fatal, but some of them are preventable. This study aimed to determine the types and frequencies of serious cutaneous ADRs and the methods employed to manage and prevent them, as well as to assess the factors related to their seriousness. **Methods:** A cross-sectional study was conducted retrospectively on inpatients and outpatients at a tertiary care hospital. All data were collected from the medical records database over a period of 3 years. Serious cutaneous ADRs were identified in the hospital database using the International Classification of Disease and Related Health Problems, 10th Revision (ICD-10). **Results:** A total of 2151 cases were retrieved using the ICD-10, and 436 patients were randomly selected for this study. Of these, 218 patients experienced ADRs (50.0%). The major clinical symptoms of the eight serious ADRs included anaphylaxis (38.5%) and urticaria (30.2%). The most commonly suspected drug group was antibiotics (45.0%). The main methods of ADR management were drug treatment (84.4%) and drug withdrawal (81.2%). The primary method of ADR prevention was patient drug allergy cards (52.3%). Factors affecting the severity of ADRs were having an underlying condition (*p* = 0.031) and the concomitant use of drugs (*p* = 0.044). **Conclusions:** Anaphylaxis was the most common serious ADR. Patients with underlying diseases and those taking concomitant drugs are more likely to present with serious ADRs. The prevention of serious ADRs should be promoted at all levels in hospitals to reduce harm and prevent their reoccurrence.

## 1. Introduction

Adverse drug reactions (ADRs) are a major public health problem [1,2]. The incidence of ADRs has been estimated to be between 6 and 10% [1,2]. A systematic review and meta-analysis showed that the pooled prevalence of ADRs in a primary care setting was 8.32% [3]. In one study, the incidence of severe ADRs was 7.01% [2]. In hospital patients, the overall incidence of serious ADRs was found to be 2.1%, with 0.19% of the ADRs being fatal [1]. The impacts of ADRs were found to result in hospitalization in 9.1% of cases, disability in 0.7%, and mortality in 0.9% [4]. Cutaneous adverse drug reactions affect approximately 2–5% of people [5,6]. A prospective study in Germany reported that the incidence of SCARs was 1.53–1.89 cases per million per year [7]. Another study estimated the incidence of SCARs to be 0.4–1.2 cases per million per year [8]. In Thailand, the mean incidence of SCARs was reported to be 5.22 cases per million per year [9]. Among hospitalized pediatric patients, the annual incidence of SCARs was 17.6 cases per million inpatients [10]. In China, the prevalence rate of SCARs was 0.32 cases per 1000 hospitalized patients [11]. However, the incidence rate of SCARs has been reported to be higher among Asian populations. Several studies using the Hartwig Scale for rated ADR severity levels found that the incidence of severe ADRs ranged from 0% to 10% [2,12,13,14,15,16]. ADRs also have significant financial impacts on healthcare systems [2,4].

In Thailand, the largest group of reported ADRs are skin and appendage disorders, which range from 30% to 40% of all reported ADRs [17]. Severe cutaneous adverse reactions (SCARs) are a group of rare but potentially life-threatening, drug-induced hypersensitivity reactions that affect the skin and, in some cases, other organs [18,19]. The mechanisms of SCARs involve a combination of genetic susceptibility, immune activation, and drug metabolism. SCARs are primarily delayed T-cell-mediated hypersensitivity reactions [18,20]. Understanding these mechanisms is critical for improving diagnosis, treatment, and prevention strategies. SCARs include drug reaction with eosinophilia and systemic symptoms (DRESS), Stevens–Johnson syndrome (SJS), toxic epidermal necrolysis (TEN), and acute generalized exanthematous pustulosis (AGEP) [19]. SCARs are life-threatening conditions that require early identification and intervention. Early identification enables the timely discontinuation of the causative drug, which is the cornerstone of treatment. Delayed diagnosis can result in worsening symptoms, severe complications, or even death [19,20]. Several tools and strategies are available to aid in the identification of SCARs. For example, scoring systems like SCORTEN assess the severity and predict mortality for SJS/TEN [21], while the RegiSCAR criteria assist in confirming the diagnosis of DRESS based on clinical and laboratory findings [22]. Current pharmacogenomic studies have made significant progress in demonstrating the relationship between human leukocyte antigen (HLA) alleles and SCARs. Numerous studies have shown a strong association between SCARs and HLA-linked genetic predispositions [18,23,24,25,26,27]. SCARs are rare, but they are serious enough to cause death [28]. The most common serious skin reactions are SJS (50.1%) and TEN (25.4%), with the leading causes being antibiotics (26.0%), sedative–hypnotics and anticonvulsants (21.6%), and antipyretics and analgesics (17.1%) [28]. A study on drug-induced ADRs and HLA allele associations revealed that several well-known drugs, such as allopurinol, carbamazepine, dapsone, and abacavir, are associated with specific HLA alleles that can induce SCARs [24].

To reduce morbidity and mortality in SCARs, early diagnosis and prompt withdrawal of the potentially causative drug are essential [10]. The major ADR management methods used by healthcare professionals (HCPs) are medicinal treatment, removal of the causative drug, management of skin or mucosal involvement, supportive care with particular focus on fluid replacement, nutrition, analgesia, and infection prevention [19,29]. Patients should be provided with sufficient education on avoiding causative medications to prevent re-exposure or recurrence [19,29]. The factors associated with the occurrence of more severe levels of ADRs were sex (females), age (elderly), and an increased number of medications [2,30,31]. Another important point is the association of HLA allele genetic polymorphisms, which can cause SCARs [27]. These variants may differ across populations [18,32]. The genetic risk factors are particularly significant, especially in SCARs induced by carbamazepine, abacavir, and allopurinol [18].

There are still only a limited number of studies on serious cutaneous ADR using data from medical records, as these data are primarily retrieved from spontaneous reporting systems. However, data from spontaneous reports are provided voluntarily by HCPs, with underreporting being a major limitation. This study provides fundamental information for HCPs and patients to recognize the types of drugs that can cause SCARs, promoting safe drug use and monitoring, reducing the risk of SCARs, and enhancing the safety of patient medication. Therefore, the current study was undertaken to determine the types and frequencies of serious cutaneous ADRs, evaluate the management and prevention methods employed by HCPs, and assess factors related to the seriousness of ADRs.

## 2. Materials and Methods

This cross-sectional study was conducted retrospectively by collecting patient data for serious cases of skin ADRs from medical records and the hospital pharmacy database at a tertiary-care teaching hospital in Northeastern Thailand for a period of 3 years (1 September 2016 to 1 September 2019). Eligible patients included both inpatients and outpatients aged 18 years or older who had experienced one of eight syndromes related to serious ADRs: angioedema (ANG), anaphylaxis (ANA), urticaria, fixed drug eruption (FDE), drug reaction with eosinophilia and systemic symptoms (DRESS), Stevens–Johnson syndrome (SJS), toxic epidermal necrolysis (TEN), and acute generalized exanthematous pustulosis (AGEP). Patients whose medical records were unavailable or incomplete were excluded from the study. All patient data related to serious skin ADRs were collected solely by the research team, without directly obtaining information from patients. The sample size calculation was based on Cochran’s sample size formula [33], assuming an average reporting proportion for SCARs of 0.26 [34] with a 5% margin of error. This resulted in a required sample size of 292 cases. To ensure an adequate sample size, a target of at least 400 patients was established.

The data collection form was designed and developed by the research team and based on the study’s objectives. The data collection form’s content was validated by two HCPs with proficiency in the field of ADRs. The data collection form consisted of the following four main sections (Supplementary File):

Part 1: demographic questions that covered gender, age, date of admission, and discharge.

Part 2: the patient’s medical history that covered drug allergies and ADR experience, including the name of the suspected drugs, indication, concomitant drugs, underlying condition, timeline, clinical symptoms, hypersensitivity type, severity assessment, and causality assessment.

Part 3: assessment of the clinical outcomes relating to death, life-threatening conditions, hospitalization, morbidity, fetal abnormalities, or no effects.

Part 4: Management of ADRs, including dechallenge, rechallenge, dose adjustments or provision of medication. Determination of the prevention strategy from the medical record, sticker chart, drug allergy card, or surveillance report was also included.

The final data collection form was used by the researchers to gather patients’ data from the medical records and the hospital pharmacy database (Praxticol^®^ and Health Object program^®^, version 3.0.440.55.9). Eight syndromes related to serious ADRs were identified using the following International Statistical Classification of Disease and Health-related Problem (ICD-10) codes: L27.0, L27.1, L50.0, L51.1, L51.2, L53.8, T78.2, T78.3, and T88.6 (DRESS, FDE, Urticaria, SJS, TEN, AGEP, ANA, ANG, and ANA, respectively). After receiving the complete list of patients according to the ICD-10, we calculated the proportion of patients in each ICD-10 category. Simple random sampling was conducted using Microsoft Excel to generate random numbers for the selection of patients and to achieve the required sample size. The patients’ hospital numbers were then recoded and kept confidential. Only the researchers had access to these data. Additional ADR information was retrieved from the medical records database.

The Hartwig Scale was used to assess the severity of the ADRs by the research pharmacists. The Hartwig Scale classifies ADRs into the following seven levels of severity: levels 1 and 2 are categorized as mild, levels 3 and 4 as moderate, and levels 5, 6, and 7 as severe. Mild ADRs are self-limiting and resolve over time without the need for treatment. Moderate ADRs require treatment or result in an increased length of hospital stay by at least one day. Severe ADRs are life-threatening, cause permanent harm, or result in death [35]. Serious ADRs were defined according to the criteria of the Health Product Vigilance Center (HPVC), including ADRs that can cause death, result in life-threatening conditions, require inpatient hospitalization, or lead to persistent or significant disability or incapacity and congenital anomaly or birth defect [36]. The causality assessment was conducted using Naranjo’s algorithm and the World Health Organization’s (WHO) criteria. The Naranjo algorithm has the following four categories: definite (score > 9), probable (score 5–8), possible (score 1–4), and doubtful (score < 0). The four WHO criteria categories are certain, probably/likely, possible, and unlikely. Patient data and ADR severity ratings were collected by two research pharmacists who were trained to use the data collection form and the Hartwig assessment tool.

Data retrieved form medical records were analyzed using IBM SPSS for Windows version 26.0. Descriptive statistics were employed to analyze the demographic data, frequencies, and types of ADRs. The Pearson’s chi-squared test and Fisher’s exact test were used for subgroup comparisons of categorical data, while multiple logistic regression was applied to identify factors associated with the seriousness of ADRs. The results with *p*-values < 0.05 were considered statistically significant. This study was approved by the Khon Kaen University Ethics Committee for Human Research (no. HE621487, on 5 November 2019).

## 3. Results

### 3.1. Demographic Data

A total of 2151 patient data were retrieved according to the ICD-10, and 436 patients were randomly selected for this study. Among these, 218 patients experienced ADRs (50.0%), 12 patients had food allergies (2.8%), and 206 patients were classified under other causes (47.2%). The patient demographics are presented in Table 1. More than half of patients were female (*n* = 126, 57.8%), and their mean age was 43.89 ± 22.55 years (range = 22–72 years). The majority of patients were inpatients who had experienced a serious skin ADR (*n* = 195, 89.4%). Half of the patients were admitted after prodrome occurred (*n* = 124, 56.9%), had one to two co-morbidities (*n* = 116, 53.2%), and were taking at least one concurrent medication (*n* = 122, 55.9%).

### 3.2. Types and Frequencies of Serious Cutaneous ADRs

The top three clinical symptoms of the eight serious ADRs were anaphylaxis (38.5%), urticaria (30.2%), and DRESS (13.8%). The proportions of the four SCARs syndromes were as follows: DRESS (13.8%), SJS (5.5%), TEN (2.3%), and AGEP (1.8%). The three drug groups causing the highest number of ADRs were antibiotics (49.1%), nonsteroidal anti-inflammatory drugs (NSAIDs) (15.1%), and chemotherapy (9.2%). Summaries of the drug groups with the top three drugs most commonly suspected of inducing SCARs are shown in Table 2.

The severity of the ADRs was reported as moderate in 8.0% of cases, with the remainder classified as severe (92.0%). All patients who were diagnosed with DRESS (*n* = 30), AGEP (*n* = 4), and TEN (*n* = 5) were classified as having severe ADRs. None of the suspected ADRs were classified as mild, according to the Hartwig Scale. The majority of patients were classified as having serious ADRs (*n* = 203, 93.1%) according to the criteria of the Health Product Vigilance Center (HPVC). All patients who were diagnosed with DRESS (*n* = 30), FDE (*n* = 8), AGEP (*n* = 4), TEN (*n* = 5), or anaphylaxis (*n* = 84) were classified as having serious ADRs. Most of the patients classified as having serious ADRs required hospitalization (*n* = 202, 92.7%) (Table 3). No deaths from SCARs were reported in this study.

Based on the Naranjo algorithm, 99 suspected cases of ADRs (45.4%) were classified as probable, 116 (53.2%) as possible, and 3 (1.4%) as doubtful or false ADRs. According to the WHO’s criteria, 90 suspected cases of ADRs (41.3%) were categorized as probable, 124 (56.9%) as possible, 1 (0.5%) as unlikely or false, and 3 (1.3%) as unassessable. However, none of the suspected ADRs were classified as definite by either method.

### 3.3. Methods of Managing and Preventing ADRs

The most common methods used by physicians in ADR management were drug treatment (84.4%) and drug withdrawal (81.2%). All patients (100%) diagnosed with FDE, SJS, TEN, and angioedema underwent drug withdrawal as a method of managing cutaneous ADRs. The top three SCARs that utilized drug treatment as a method of managing cutaneous ADRs were TEN (100%), DRESS (93.3%), and anaphylaxis (90.5%). The top three drugs used to treat serious ADRs were systemic antihistamine (75.5%), systemic corticosteroid (46.7%), and adrenaline (39.1%). Patients who were diagnosed with FDE, SJS, TEN, and angioedema were found to have the highest proportion of drug withdrawal (100.0%). Patients diagnosed with AGEP were found to have the highest proportion of continued use of the suspected drug (50.0%). Almost one-fifth of patients continued to use suspected drugs (17.0%), with almost two-thirds of patients continuing the drug at the same dose (67.6%). Significant differences in clinical symptoms were found for both ADR management methods, including drug withdrawal and continued use of the suspected drugs (*p* < 0.001 and *p* = 0.003). The subgroups of the ADR management method in case of continued used of the suspected drug exhibited significant differences in clinical symptoms (*p* < 0.001) (Table 4).

The methods of preventing ADR used by HCPs included providing patients with drug allergy cards (52.3%), recording ADRs in medical records (35.3%), ADR education for patients (26.1%), and using stickers on medical charts (6.0%). There were significant differences in clinical symptoms related to the various methods of ADR prevention, with only the provision of drug allergy cards exhibiting a significant preventive effect on clinical symptoms (*p* = 0.006) (Table 4).

### 3.4. Factors Related to the Seriousness of the ADRs

A univariate analysis of the factors related to the seriousness of the ADRs revealed that male patients (*p* = 0.033), those with underlying diseases (*p* = 0.001), and concomitant drugs (*p* = 0.004) were all significantly associated with the seriousness of the ADRs. A multiple logistic regression showed the factors independently associated with patient confidence having underlying diseases (OR 10.23; 95% CI 1.23, 85.05; *p* = 0.031) and concomitant drugs (OR 4.08; 95% CI 1.04, 16.03; *p* = 0.044) (Table 5).

## 4. Discussion

This study sought to determine the types and frequencies of angioedema, anaphylaxis, urticaria, fixed drug eruption, and SCARs in a large university hospital in Northeastern Thailand. We found that the drug group that caused the highest number of ADRs was antibiotics. The most common methods of managing ADRs used by physicians were drug treatment and drug withdrawal, and the most common method of preventing ADRs used by HCPs was the provision of drug allergy cards to patients. The types and frequencies of the ADRs studied at the hospital are different from previously published studies, which can be explained by differences in population and the scope of suspected ADRs chosen. Multivariate analysis showed that having an underlying disease and receiving concomitant drugs were associated with serious ADRs.

In relation to the SCAR syndromes most frequently associated with suspected ADRs, the results of this study are consistent with those of previous studies [19,28,37,38,39]. However, different proportions of each SCAR syndrome were found among the studies due to the differences in their scopes. In this study, the most frequent clinical symptoms of serious ADRs were anaphylaxis (38.5%), urticaria (30.2%), and DRESS (13.8%). This is different than in previous studies, which found that the most common clinical symptoms were maculopapular rash, fixed drug eruption, and urticaria [12,40,41]. Previous studies that used the Thai Vigibase and the FDA Adverse Event Reporting System (FAERS) have shown that the most frequent clinical types of SCARs were SJS and TEN [42,43]. However, this study demonstrates that DRESS is the most common type of SCAR. Additionally, our study revealed that antibiotics were the drug group most commonly associated with SCARs, consistent with previous studies reporting that antibiotics accounted for the majority of SCAR cases (approximately 20–30%) [28,42,44]. Notably, this study observed a higher proportion of antibiotic-induced SCARs (49.1%). NSAIDs and chemotherapy agents were identified as the next most common drug groups causing SCARs, which aligns with the findings of other studies [42,44].

The current study confirmed the findings of previous research, highlighting the role of antibiotics as the most common cause of ADRs reported in the literature [2,12,28,31,41,45,46]. The drugs most frequently causing anaphylaxis were antibiotics (32.1%), NSAIDs (27.4%), and chemotherapy (9.5%), contrary to Kemp et al., who found NSAIDs (51.9%) to be the most common cause of anaphylaxis, followed by antibiotics (23.1%) [47]. The drugs most commonly reported to cause SJS and TEN in this study were antibiotics (41.7% and 60.0%), anti-gout (16.7% and 20.0%), and antiepileptics (8.3% and 20.0%). The healthcare system in the study’s hospital, which is a tertiary-care teaching hospital, may be unrepresentative of other hospitals. Some differences in drug usage, such as for imatinib, were reported, which is due to this drug being available at the study’s hospital. However, the majority of prescribed drugs at the study’s hospital were included in the National Drug Formulary, aligning with economic conditions. This study found that the most common drugs suspected of causing serious ADRs were ceftriaxone, allopurinol, and phenytoin, all of which are listed in the National Drug Formulary. Additionally, a later period of the study coincided with the COVID-19 pandemic, which may have affected prescribing patterns and patient care. Prior studies have reported allopurinol (14.2%) and carbamazepine (7.1%) as the most common drugs associated with SJS or TEN [48]. Recent pharmacogenomic studies showed that HLA-B*15:02 is strongly linked to carbamazepine-induced SJS/TEN in Asians [23,27,49], while HLA-B*58:01 is associated with allopurinol-induced SCARs in Han Chinese and Thai populations [25,26]. This may reflect differences in drug utilization in differing healthcare systems and the management of the risk of ADRs with different drugs.

The diagnosis of a severe or serious ADR requires the collection of a detailed medical history, including medications used over several months, a history of drug allergies, a family history of drug reactions, and any history of skin diseases. Re-enforcing previous studies, this study shows that the primary methods of managing ADRs used by physicians were drug treatment and drug withdrawal, with the most common treatments for serious ADRs being antihistamines, steroids, and adrenaline [19,29].

The current study shows that the methods for preventing ADRs implemented by HCPs included providing patients with drug allergy cards, recording ADRs in medical records, educating patients on ADRs, and attaching stickers to medical charts. This corresponds with previous studies indicating the methods used to prevent ADRs, which included providing counseling, educational programs about avoiding causative drugs, and helpful tools [12,19,29]. Additionally, we found properly recording a patient’s history in their medical record and provision of drug allergy cards to patients were also used [50]. In some cases, ADRs can be prevented by providing patient education, informative materials, and helpful tools, such as drug alert cards for patients with emergency telephone numbers, which may prevent future harm [51,52].

The data used in this study did not include details about ADR prevention related to the use of genetic testing to screen for SCARs during the study period. In Thailand, genetic testing to screen for SCARs covered under universal healthcare was approved around 2018 for carbamazepine and in 2021 for allopurinol. Recent pharmacogenomic studies have demonstrated a strong association between SCARs and specific HLA alleles. Genetic testing for HLA-B*58:01 before initiating allopurinol is considered highly cost-effective in Thailand [53]. Genetic testing may enhance drug safety by reducing the risk of SCARs. However, several problems have been identified with the use of genetic testing for screening, including the high costs, limitations of the tests, and delays in receiving the results [20]. Therefore, one study aimed to utilize non-genetic risk factors to develop a model for predicting severe allopurinol hypersensitivity [54].

The current study shows that patients with co-morbidities and co-medication were significantly more likely to present with serious ADRs, which is in line with previous research [2,30,31,46,55,56,57,58]. However, previous studies have suggested that advanced age is associated with severe or serious ADRs [2,46,55,58]. Not unexpectedly, higher levels of ADR ‘bothersomeness’ and its disturbance of daily life are associated with more severe ADRs [16,57].

A major strength of this study is that it was conducted at a tertiary-care teaching hospital in Northeastern Thailand, which has a large number of patients and a greater variety of drug-use regimens, increasing the chances of encountering ADRs. A limitation of this study is that it is a retrospective study that collected data from patients’ medical records, which may result in some of the data being incomplete. Additionally, since the study was conducted in a single tertiary hospital in Northeastern Thailand, the findings may not be fully generalizable to other regions of Thailand or to international contexts.

## 5. Conclusions

The most frequent clinical symptom of cutaneous ADRs was anaphylaxis, with antibiotics, NSAIDs, and chemotherapy being the drug groups most involved in cutaneous ADRs. Almost all patients with serious ADRs required hospitalization. Stopping use of the suspected drugs and providing supportive medical treatment were the major methods used in the management of serious ADRs. Provision of drug allergy cards was the primary method used to prevent future serious ADRs. Patients with underlying diseases and those taking concomitant drugs were significantly associated with serious ADRs.

Serious ADRs have low incidence but require hospitalization and can significantly impact human life, making HCP awareness of them essential for their diagnosis, management, and prevention. Preventing serious ADRs is crucial for reducing harm. This can be achieved by maintaining a proper medical history, prescribing appropriate drugs, counseling and educating patients about avoiding causative medications, and providing helpful tools. Additionally, implementing genetic testing to screen for SCARs in patients receiving high-risk drugs can provide significant benefits toward the prevention of SCARs.

## Figures and Tables

**Table 1:** Demographic characteristics of the patients (*n* = 218).

Demographic Data	No. (%)								
	DRESS (*n* = 30)	FDE (*n* = 8)	AGEP (*n* = 4)	SJS (*n* = 12)	TEN (*n* = 5)	ANA (*n* = 84)	ANG (*n* = 9)	Urticaria (*n* = 66)	Total (*n* = 218)
Age (years)									
≤45	15 (50.0)	3 (37.5)	2 (50.0)	5 (41.7)	1 (20.0)	37 (44.0)	6 (66.7)	34 (51.5)	103 (47.2)
46–60	7 (23.3)	4 (50.0)	1 (25.0)	6 (50.0)	2 (40.0)	21 (25.0)	1 (11.1)	18 (27.3)	60 (27.5)
>60	8 (26.7)	1 (12.5)	1 (25.0)	1 (8.3)	2 (40.0)	26 (31.0)	2 (22.2)	14 (21.2)	55 (15.2)
Mean ± SD	40.97 ± 29.90	50.13 ± 22.48	45.00 ± 12.96	42.08 ± 20.95	59.60 ± 21.09	44.80 ± 22.59	36.89 ± 27.85	43.35 ± 22.28	43.89 ± 22.55
Gender									
Male	20 (66.7)	4 (50.0)	2 (50.0)	8 (66.7)	2 (40.0)	30 (35.7)	2 (22.2)	24 (36.4)	92 (42.2)
Female	10 (33.3)	4 (50.0)	2 (50.0)	4 (33.3)	3 (60.0)	54 (64.3)	7 (77.8)	42 (63.6)	126 (57.8)
Occupation									
Public servant	6 (20.0)	1 (12.5)	1 (25.0)	0 (0.0)	1 (20.0)	5 (6.0)	0 (0.0)	5 (7.6)	19 (8.7)
Agriculture	3 (10.0)	1 (12.5)	3 (75.0)	3 (25.0)	1 (20.0)	6 (7.1)	0 (0.0)	7 (10.6)	24 (11.0)
Officer, Mercenary	3 (10.0)	0 (0.0)	0 (0.0)	0 (0.0)	1 (20.0)	3 (3.6)	0 (0.0)	4 (6.1)	11 (5.0)
Not working	7 (23.3)	0 (0.0)	0 (0.0)	2 (16.7)	1 (20.0)	25 (29.8)	2 (22.2)	13 (19.7)	50 (22.9)
Others	11 (36.7)	6 (75.0)	0 (0.0)	7 (58.3)	1 (20.0)	45 (53.6)	7 (77.8)	37 (56.1)	114 (52.3)
Type of patient									
OPD	0 (0.0)	1 (12.5)	0 (0.0)	2 (16.7)	1 (20.0)	0 (0.0)	8 (88.9)	11 (16.7)	23 (10.6)
IPD	30 (100.0)	7 (87.5)	4 (100.0)	10 (83.3)	4 (80.0)	84 (100.0)	1 (11.1)	55 (83.3)	195 (89.4)
Onset of ADRs									
ADR-related admission	21 (70.0)	4 (50.0)	2 (50.0)	10 (83.3)	5 (100.0)	60 (71.4)	8 (88.9)	14 (21.2)	124 (56.9)
ADRs occurred during hospital stay	9 (30.0)	4 (50.0)	2 (50.0)	2 (16.7)	0 (0.0)	24 (28.6)	1 (11.1)	52 (78.8)	94 (43.1)
Number of concomitant diseases									
0	1 (3.3)	2 (25.0)	0 (0.0)	5 (41.7)	1 (20.0)	39 (46.4)	6 (66.7)	20 (30.3)	74 (33.9)
1–2	23 (76.7)	4 (50.0)	3 (75.0)	3 (25.0)	2 (40.0)	37 (44.0)	3 (33.3)	41 (62.1)	116 (53.2)
>2	6 (3.9)	2 (25.0)	1 (25.0)	4 (33.3)	2 (40.0)	8 (9.5)	0 (0.0)	5 (7.6)	28 (12.8)
Number of concomitant drugs									
0	9 (30.0)	2 (25.0)	1 (25.0)	4 (33.3)	2 (40.0)	47 (56.0)	8 (88.9)	23 (34.8)	96 (44.0)
1–2	8 (26.7)	3 (37.5)	2 (50.0)	2 (16.7)	1 (20.0)	16 (19.0)	0 (0.0)	13 (19.7)	45 (20.6)
>2	13 (43.3)	3 (37.5)	1 (25.0)	6 (50.0)	2 (40.0)	21 (25.0)	1 (11.1)	30 (45.5)	77 (35.3)

ANA: anaphylaxis; ANG: angioedema; DRESS: Drug Rash with Eosinophilia and Systemic Symptoms; FDE: fixed drug eruption; AGEP: acute generalized exanthematous pustulosis; SJS: Stevens–Johnson syndrome; TEN: toxic epidermal necrolysis; OPD: outpatient department; IPD: inpatient department; No.: number.

**Table 2:** Drug groups with the top 3 drugs most commonly suspected of inducing SCARs (*n* = 218).

Drug Group	No. (%)								Total (*n* = 218)
	DRESS (*n* = 30)	FDE (*n* = 8)	AGEP (*n* = 4)	SJS (*n* = 12)	TEN (*n* = 5)	ANA (*n* = 84)	ANG (*n* = 9)	Urticaria (*n* = 66)	
Antibiotics	19 (63.3)	2 (25.0)	3 (75.0)	6 (50.0)	3 (60.0)	27 (32.1)	2 (22.2)	45 (68.2)	107 (49.1)
Ceftriaxone	2 (6.7)	1 (12.5)	1 (25.0)	0 (0.0)	0 (0.0)	8 (9.5)	1 (11.1)	8 (12.1)	21 (9.6)
Ciprofloxacin	1 (3.3)	0 (0.0)	0 (0.0)	0 (0.0)	0 (0.0)	4 (4.8)	0 (0.0)	4 (6.1)	9 (4.1)
Clindamycin	0 (0.0)	0 (0.0)	0 (0.0)	0 (0.0)	0 (0.0)	4 (4.8)	0 (0.0)	5 (7.6)	9 (4.1)
Antiepileptics	7 (23.3)	2 (25.0)	0 (0.0)	1 (8.3)	1 (20.0)	2 (2.4)	1 (11.1)	2 (3.0)	16 (7.3)
Phenytoin	5 (16.7)	0 (0.0)	0 (0.0)	1 (8.3)	1 (20.0)	0 (0.0)	0 (0.0)	1 (1.5)	8 (3.7)
Carbamazepine	1 (3.3)	1 (12.5)	0 (0.0)	0 (0.0)	0 (0.0)	2 (2.4)	0 (0.0)	0 (0.0)	4 (1.8)
Phenobarbital	1 (3.3)	0 (0.0)	0 (0.0)	0 (0.0)	0 (0.0)	0 (0.0)	0 (0.0)	0 (0.0)	1 (0.5)
Anti-gout	2 (6.7)	0 (0.0)	0 (0.0)	2 (16.7)	1 (20.0)	0 (0.0)	0 (0.0)	0 (0.0)	5 (2.3)
Allopurinol	2 (6.7)	0 (0.0)	0 (0.0)	2 (16.7)	1 (20.0)	0 (0.0)	0 (0.0)	0 (0.0)	5 (2.3)
Anti-muscarinic	0 (0.0)	0 (0.0)	0 (0.0)	0 (0.0)	0 (0.0)	1 (1.2)	0 (0.0)	1 (1.5)	2 (0.9)
Flavoxate	0 (0.0)	0 (0.0)	0 (0.0)	0 (0.0)	0 (0.0)	0 (0.0)	0 (0.0)	1 (1.5)	1 (0.5)
Hyoscine	0 (0.0)	0 (0.0)	0 (0.0)	0 (0.0)	0 (0.0)	1 (1.2)	0 (0.0)	0 (0.0)	1 (0.5)
Chemotherapy	1 (3.3)	2 (25.0)	0 (0.0)	0 (0.0)	0 (0.0)	8 (9.5)	0 (0.0)	9 (13.6)	20 (9.2)
Oxaliplatin	0 (0.0)	0 (0.0)	0 (0.0)	0 (0.0)	0 (0.0)	4 (4.8)	0 (0.0)	4 (6.1)	8 (3.7)
Paclitaxel	0 (0.0)	0 (0.0)	0 (0.0)	0 (0.0)	0 (0.0)	2 (2.4)	0 (0.0)	3 (4.5)	5 (2.3)
Etoposide	0 (0.0)	0 (0.0)	0 (0.0)	0 (0.0)	0 (0.0)	1 (1.2)	0 (0.0)	1 (1.5)	2 (0.9)
Immunosuppressants	0 (0.0)	0 (0.0)	1 (25.0)	0 (0.0)	0 (0.0)	1 (1.2)	0 (0.0)	0 (0.0)	2 (0.9)
Azathioprine	0 (0.0)	0 (0.0)	1 (25.0)	0 (0.0)	0 (0.0)	0 (0.0)	0 (0.0)	0 (0.0)	1 (0.5)
Rituximab	0 (0.0)	0 (0.0)	0 (0.0)	0 (0.0)	0 (0.0)	1 (1.2)	0 (0.0)	0 (0.0)	1 (0.5)
NSAIDs	0 (0.0)	1 (12.5)	0 (0.0)	1 (8.3)	0 (0.0)	23 (27.4)	4 (44.4)	4 (6.1)	33 (15.1)
Diclofenac	0 (0.0)	0 (0.0)	0 (0.0)	0 (0.0)	0 (0.0)	9 (10.7)	1 (11.1)	0 (0.0)	10 (4.6)
Ibuprofen	0 (0.0)	0 (0.0)	0 (0.0)	1 (8.3)	0 (0.0)	7 (8.3)	1 (11.1)	1 (1.5)	10 (4.6)
Piroxicam	0 (0.0)	1 (12.5)	0 (0.0)	0 (0.0)	0 (0.0)	3 (3.6)	0 (0.0)	0 (0.0)	4 (1.8)
Opiate	0 (0.0)	0 (0.0)	0 (0.0)	0 (0.0)	0 (0.0)	2 (2.4)	0 (0.0)	1 (1.5)	3 (1.4)
Fentanyl	0 (0.0)	0 (0.0)	0 (0.0)	0 (0.0)	0 (0.0)	1 (1.2)	0 (0.0)	0 (0.0)	1 (0.5)
Morphine	0 (0.0)	0 (0.0)	0 (0.0)	0 (0.0)	0 (0.0)	0 (0.0)	0 (0.0)	1 (1.5)	1 (0.5)
Tramadol	0 (0.0)	0 (0.0)	0 (0.0)	0 (0.0)	0 (0.0)	1 (1.2)	0 (0.0)	0 (0.0)	1 (0.5)
Psychiatric drugs	0 (0.0)	0 (0.0)	0 (0.0)	0 (0.0)	0 (0.0)	1 (1.2)	0 (0.0)	0 (0.0)	1 (0.5)
Fluoxetine	0 (0.0)	0 (0.0)	0 (0.0)	0 (0.0)	0 (0.0)	1 (1.2)	0 (0.0)	0 (0.0)	1 (0.5)
Others	3 (10.0)	0 (0.0)	0 (0.0)	0 (0.0)	0 (0.0)	19 (22.6)	4 (44.4)	3 (4.5)	29 (13.3)
SPEEDA^®^	0 (0.0)	0 (0.0)	0 (0.0)	0 (0.0)	0 (0.0)	3 (3.6)	0 (0.0)	0 (0.0)	3 (1.4)
Lidocaine	0 (0.0)	0 (0.0)	0 (0.0)	0 (0.0)	0 (0.0)	1 (1.2)	0 (0.0)	1 (1.5)	2 (0.9)
Omeprazole	0 (0.0)	0 (0.0)	0 (0.0)	0 (0.0)	0 (0.0)	2 (2.4)	0 (0.0)	0 (0.0)	2 (0.9)

ANA: anaphylaxis; ANG: angioedema; DRESS: Drug Rash with Eosinophilia and Systemic Symptoms; FDE: fixed drug eruption; AGEP: acute generalized exanthematous pustulosis; SJS: Stevens–Johnson syndrome; TEN: toxic epidermal necrolysis; No.: number; NSAIDs: nonsteroidal anti-inflammatory drugs.

**Table 3:** Classification of the reported SCARs based on the severity level and seriousness of the ADRs.

**Severity Level**	**No. (%)**								**Total** **(*n* = 212)**
	**Hypersensitivity Type 4**					**Hypersensitivity Type 1**			
	**DRESS** **(*n* = 30)**	**FDE** **(*n* = 8)**	**AGEP** **(*n* = 4)**	**SJS** **(*n* = 12)**	**TEN** **(*n* = 5)**	**ANA** **(*n* = 80)**	**ANG** **(*n* = 8)**	**Urticaria** **(*n* = 65)**	
Moderate	0 (0.0)	1 (12.5)	0 (0.0)	2 (16.7)	0 (0.0)	1 (1.2)	4 (50.0)	9 (13.8)	17 (8.0)
Severe	30 (100)	7 (87.5)	4 (100)	10 (83.3)	5 (100)	79 (98.8)	4 (50.0)	56 (86.2)	195 (92.0)
**Seriousness Level**	**No. (%)**								**Total** **(*n* = 218)**
	**Hypersensitivity Type 4**					**Hypersensitivity Type 1**			
	**DRESS** **(*n* = 30)**	**FDE** **(*n* = 8)**	**AGEP** **(*n* = 4)**	**SJS** **(*n* = 12)**	**TEN** **(*n* = 5)**	**ANA** **(*n* = 84)**	**ANG** **(*n* = 9)**	**Urticaria** (***n* = 66)**	
Serious	30 (100.0)	8 (100.0)	4 (100.0)	10 (83.3)	5 (100.0)	84 (100.0)	6 (66.7)	56 (84.8)	203 (93.1)
Life-Threatening	1 (3.3)	0 (0.0)	0 (0.0)	0 (0.0)	0 (0.0)	0 (0.0)	0 (0.0)	0 (0.0)	1 (0.5)
Hospitalization	29 (96.7)	8 (100)	4 (100)	10 (83.3)	5 (100)	84 (100)	6 (66.7)	56 (84.8)	202 (92.7)
Non-serious	0 (0.0)	0 (0.0)	0 (0.0)	2 (16.7)	0 (0.0)	0 (0.0)	3 (33.3)	10 (15.2)	15 (6.9)

ANA: anaphylaxis; ANG: angioedema; DRESS: Drug Rash with Eosinophilia and Systemic Symptoms; FDE: fixed drug eruption; AGEP: acute generalized exanthematous pustulosis; SJS: Stevens–Johnson syndrome; TEN: toxic epidermal necrolysis; No.: number.

**Table 4:** The methods of ADR management and prevention in relation to the type of ADR (*n* = 218).

**Management**	**No. (%)**								**Total** **(*n* = 218)**	***p*-Value ^a^**
	**Hypersensitivity Type 4**					**Hypersensitivity Type 1**				
	**DRESS** **(*n* = 30)**	**FDE** **(*n* = 8)**	**AGEP** **(*n* = 4)**	**SJS** **(*n* = 12)**	**TEN** **(*n* = 5)**	**ANA** **(*n* = 84)**	**ANG** **(*n* = 9)**	**Urticaria** (***n* = 66)**		
Stopping use of suspected drugs	27 (90.0)	8 (100.0)	2 (50.0)	12 (100.0)	5 (100.0)	77 (91.7)	9 (100.0)	37 (56.1)	177 (81.2)	<0.001 ^b^*
Continued use of suspected drugs	2 (6.7)	0 (0.0)	2 (50.0)	0 (0.0)	0 (0.0)	13 (15.5)	0 (0.0)	20 (30.3)	37 (17.0)	0.003 ^b^*
Same dose	2 (100.0)	0 (0.0)	2 (100.0)	0 (0.0)	0 (0.0)	3 (23.1)	0 (0.0)	18 (90.0)	25 (67.6)	<0.001 ^b^*
Change of administration methods	0 (0.0)	0 (0.0)	0 (0.0)	0 (0.0)	0 (0.0)	10 (76.9)	0 (0.0)	2 (10.0)	12 (32.4)	
Medical treatment	28 (93.3)	6 (75.0)	1 (25.0)	9 (75.0)	5 (100.0)	76 (90.5)	7 (77.8)	52 (78.8)	184 (84.4)	0.012 ^b^*
Adrenaline	1 (3.6)	0 (0.0)	0 (0.0)	1 (11.1)	0 (0.0)	68 (89.5)	1 (14.3)	1 (1.9)	72 (39.1)	<0.001 ^b^*
Oral antihistamine	12 (42.9)	2 (33.3)	1 (100.0)	2 (22.2)	2 (40.0)	1 (1.3)	4 (57.1)	15 (28.8)	39 (21.2)	<0.001 ^b^*
Systemic antihistamine	11 (39.3)	4 (66.7)	1 (100.0)	3 (33.3)	1 (20.0)	73 (96.1)	5 (71.4)	41 (78.8)	139 (75.5)	<0.001 ^b^*
Oral corticosteroid	9 (32.1)	2 (33.3)	0 (0.0)	4 (44.4)	3 (60.0)	3 (3.9)	0 (0.0)	3 (5.8)	24 (13.0)	<0.001 ^b^*
Systemic corticosteroid	3 (10.7)	2 (33.3)	0 (0.0)	5 (55.5)	1 (20.0)	69 (90.8)	2 (28.6)	4 (7.7)	86 (46.7)	<0.001 ^b^*
Topical corticosteroid	7 (25.0)	2 (33.3)	0 (0.0)	3 (33.3)	1 (20.0)	0 (0.0)	0 (0.0)	2 (3.8)	15 (8.2)	<0.001 ^b^*
**Prevention**	**No. (%)**								**Total** **(*n* = 218)**	***p*-Value ^a^**
	**Hypersensitivity Type 4**					**Hypersensitivity Type 1**				
	**DRESS** **(*n* = 30)**	**FDE** **(*n* = 8)**	**AGEP** **(*n* = 4)**	**SJS** **(*n* = 12)**	**TEN** **(*n* = 5)**	**ANA** **(*n* = 84)**	**ANG** **(*n* = 9)**	**Urticaria** (***n* = 66)**		
Medical record	9 (30.0)	2 (25.0)	1 (25.0)	10 (83.3)	1 (20.0)	31 (36.9)	7 (77.8)	16 (24.2)	77 (35.3)	0.190
Patient education	4 (13.3)	1 (12.5)	0 (0.0)	3 (25.0)	5 (100)	33 (39.3)	0 (0.0)	11 (16.7)	57 (26.1)	0.784
Drug allergy card	27 (90.0)	7 (87.5)	3 (75.0)	10 (83.3)	1 (20.0)	46 (54.8)	6 (66.7)	44 (66.7)	114 (52.3)	0.006 *
Sticker chart	4 (13.3)	0 (0.0)	0 (0.0)	0 (0.0)	1 (20.0)	3 (3.6)	0 (0.0)	5 (7.6)	13 (6.0)	0.179 ^b^

^a^ Pearson’s chi-square test. ^b^ Fisher’s exact test. * Significant difference at the level of <0.05. ANA: anaphylaxis; ANG: angioedema; DRESS: Drug Rash with Eosinophilia and Systemic Symptoms; FDE: fixed drug eruption; AGEP: acute generalized exanthematous pustulosis; SJS: Stevens–Johnson syndrome; TEN: toxic epidermal necrolysis; No.: number.

**Table 5:** Multiple logistic regression analysis of the factors affecting the occurrence of serious ADRs (*n* = 218).

Variable	No. (%)		Adjusted OR	95% CI		*p*-Value
	Non-Serious (*n* = 15)	Serious (*n* = 203)		Upper	Lower	
Gender						
Male	2 (13.3)	90 (44.3)	1			
Female	13 (86.7)	113 (55.7)	0.291	0.061	1.392	0.122
Co-morbidity						
No	12 (80.0)	62 (30.5)	1			0.031 *
Yes	3 (20.0)	141 (69.5)	10.230	1.230	85.045	
Co-medication						
No	14 (93.3)	82 (40.4)	1			0.044 *
Yes	1 (6.7)	121 (59.6)	4.082	1.039	16.034	

The variables included in the multiple logistic regression analysis were gender, co-morbidity, and co-medication. * Significant difference at the level of <0.05. No.: number.

## Data Availability

The data presented in this study are available upon request from the corresponding author.

## Supplementary Materials

The following supporting information can be downloaded at: https://www.mdpi.com/article/10.3390/jcm14030857/s1. Supplementary File: Serious Adverse Drug Reaction (ADR) Record Form.
