# Supplementary material for: Drug-Induced Serious Cutaneous Reactions in Hospitalized Patients: A Cross-Sectional Study
Source: J Clin Med. 2025 Jan 28;14(3):857. doi: 10.3390/jcm14030857 (PMC11818606; doi:10.3390/jcm14030857)
Supplement: Supplementary file 1 [file jcm-14-00857-s001.zip › jcm-3403893-supplementary.pdf]

Code: ________________

Serious Adverse Drug Reaction (ADR) Record Form

Part 1: Demographic information

1. Gender ☐ Male ☐ Female
2. Age ____________________ years
3. Occupation ____________________
4. Type of patients ☐ Outpatient (OPD) ☐ Inpatient (IPD)
5. Date of admission ____________ Date of discharge ____________

Part 2: Patients' medical history

CC: ____________________

HPI: ____________________

PMH: ____________________

FH: ____________________

SH: ____________________

ALL: ____________________

Physical exam: BP:__________ T:__________ RR:__________ HR:__________

____

____

____

Clinical laboratory data

Date	Lab	Normal values	Laboratory results	Interpretation	Comment

Diagnosis: ____

Drug allergy card: ( ) Suspected drug (specify)______________________________

( ) Food allergies (specify) ______________________________

( ) Other (Specify) ______________________________

Suspected drug name caused SCARs: ________________ Indication: ________________

Dose: ________________ Regimen:______________________________

Patient's past drug allergy history: ( ) No ( ) Yes

If yes, is the current cause due to the same drug?" ( ) Yes ( ) No

Drug name ________________ Symptom________________

Drug name ________________ Symptom________________

Drug name ________________ Symptom________________

( ) True allergy

( ) Pseudo allergy

( ) NSAIDs ( ) ACEI ( ) Opiate ( ) Vancomycin ( ) Protamine

( ) Other (Specify) ______________________

Patient's underlying condition or disease status:

1. ______________________ Date of diagnosis________________
2. ______________________ Date of diagnosis________________
3. ______________________ Date of diagnosis________________

Date of ADR occurrence: ________________

Date when symptoms resolved or improved: ________________

Other concomitant medications

Drug name	Dose and regimen	Duration of drug use		comment
		Start	Stop	

Information related to the patient's adverse drug reaction (ADR) occurrence

1. Type of hypersensitivity

( ) Anaphylactic type ( ) Cytotoxic type ( ) Immune complex type ( ) Delayed type

2. Urticaria assessment

( ) Wheal ผื่นลมพิษ ( ) Itching ( ) Urticarial rash

( ) Associated with the occurrence of anaphylaxis/angioedema/serum sickness

( ) Other (Specify) ____________________

3. Angioedema assessment

( ) swelling of loose connective tissue

( ) face ( ) lip ( ) tongue ( ) throat ( ) larynx ( ) Uvula ( ) Genitalia ( ) Bowel wall

( ) Colicky abdominal pain ( ) Associated with the occurrence of allergic reactions

( ) Other (Specify) ____________________

4. Anaphylactic type assessment

( ) Prodrome ( ) Similar angioedema ( ) nausea/vomiting

( ) Hypotension (Systolic pressure < 90 mmHg) ( ) syncope

( ) Bronchospasm ( ) Wheezing ( ) Dyspnea ( ) Onset within 1 hour

( ) Other (Specify) ____________________

5. Steven-Johnson syndrome (SJS) และ Toxic epidermal necrosis (TEN) assessment

( ) Prodrome ( ) fever (T>38 ℃) ( ) rash

( ) Peeling skin ( ) SJS: <10% ( ) TEN: >30% ( ) SJS/TEN: >10% - <30%

( ) Nikolsky's sign ( ) Pneumonia ( ) Dysphagia ( ) hepatitis

( ) Corneal ulcer ( ) Conjunctivitis ( ) Peptic ulcer ( ) Myocarditis

( ) Kidney failure ( ) nausea/vomiting

( ) Shedding of the mucosa ( ) mouth ( ) nose ( ) Urinary tract ( ) Vaginas ( ) Anus

( ) Other (Specify) ____________________

6. Drug reaction with eosinophilia and systemic symptoms (DRESS) assessment

( ) fever (T>38 ℃) ( ) morbiliform eruption with face edema ( ) exfoliative dermatitis

( ) lymph node enlargement ( ) arthritis ( ) hepatitis ( ) kidney impairment

( ) Eosinophilia >700 cells per µL ( ) Atypical lymphocytes ( ) Elevated transaminase

( ) Other (Specify) ____________________

7. Acute generalized exanthematous pustulosis (AGEP) assessment

( ) ไข้ (T>38 ℃) ( ) Nikolsky's sign ( ) Leukocytosis ( ) Mild eosinophilia

( ) Neutrophil count >7000/microL ( ) kidney impairment

( ) Other (Specify) ____________________

8. Fixed drug eruption assessment

( ) pruritus ( ) burning sensation ( ) Well-defined, round or oval lesions

( ) Dark pigmentation ( ) Blisters ( ) Erythema and edema

( ) no other systemic symptoms associated

( ) Other (Specify) ____________________

Hartwig's Severity Assessment Scale

Detail	Yes	No	No data
An ADR occurred but required no change in treatment with the suspected drug.			
The ADR required that treatment with the suspected drug be held, discontinued, or otherwise changed. No antidote or other treatment required. No increase in LOS.			
The ADR required that treatment with the suspected drug be held, discontinued, or otherwise changed. AND/OR An antidote or other treatment was required. No increase in LOS.			
ADR which increases LOS by at least t day OR The ADR was the reason for admission.			
ADR which requires intensive medical care.			
The adverse reaction caused permanent harm to the patient.			
The adverse reaction either directly or indirectly led to the death of the patient.			

( ) Level 1 ( ) Level 2 ( ) Level 3 ( ) Level 4 ( ) Level 5 ( ) Level 6 ( ) Level 7

Level of ADR severity: ( ) Mild (Level 1-2) ( ) Moderate (Level 3-4) ( ) Severe (Level 5-7)

Assessment of serious ADRs according to the Ministry of Public Health

( ) Serious

( ) Death

( ) Life-threatening conditions

( ) Requires inpatient hospitalization

( ) Persistent or significant disability

( ) Other (Specify) ____________________

( ) Non-serious

Causality assessment - Naranjo's algorithm

Suspected drug ______________________________ **Unusual symptoms**_____________________________

Question	Yes	No	Do not know	score
1. Are there previous conclusive reports on this reaction?	+1	0	0	
2. Did the adverse event appear after the suspected drug was administered?	+2	-1	0	
3. Did the adverse reaction improve when the drug was discontinued or a specific antagonist was administered?	+1	0	0	
4. Did the adverse reaction reappear when the drug was readministered?	+2	-1	0	
5. Are there alternative causes (other than the drug) that could on their own have caused the reaction?	-1	+2	0	
6. Did the reaction reappear when a placebo was given?	-1	+1	0	
7. Was the drug detected in the blood (or other fluids) in concentrations known to be toxic?	+1	0	0	
8. Was the reaction more severe when the dose was increased, or less severe when the dose was decreased?	+1	0	0	
9. Did the patient have a similar reaction to the same or similar drugs in any previous exposure?	+1	0	0	
10. Was the adverse event confirmed by any objective evidence?	+1	0	0	
**Total score**				

Score level: ( ) Definite (>9) ( ) Probable (5-8) ( ) Possible (1-4) ( ) Unlikely (≤0)

WHO's criteria

Suspected drug ______________________________ **Unusual symptoms**_____________________________

Detail	certain	Probable	Possible	Unlikely	No data
Time relationship					
No alternative causes					
Dechallenge					
Rechallenge					

( ) Certain ( ) Probable ( ) Possible ( ) Unlikely ( ) Unclassified ( ) Unassessable

Part 3: Assess clinical outcome after an ADR occurs

( ) ADRs which cause death ( ) Fully recovered

( ) Partially improved ( ) Improve but not fully recovered

( ) Symptom still appear ( ) Other (Specify) ____

Part 4: ADR management and prevention

4.1 ADR management methods

( ) Dechallenge Date____________________

[ ] Significant improvement in symptoms [ ] No improvement in symptoms [ ] Not known

( ) Continue using the suspected drug

[ ] Continue same dose [ ] Decrease dose (specify dose)_____________________________

[ ] Change drug administration [ ] Other (Specify) ____

( ) Rechallenge Date____________________

[ ] Rechallenge - same dose [ ] Rechallenge - decrease dose (Specify dose)________________

[ ] Other (Specify) ____

[ ] Reappeared when the drug was re-administered [ ] No symptoms occurred again [ ] Not known

( ) Medical treatment

Drug name	Dosage regimen	Treatment outcome	Comment

( ) Other (Specify) ____

4.2 ADR prevention methods

( ) Medical record/ Computer popup ( ) Report to HPVC ( ) Patient education

( ) Provide drug allergy card ( ) Attach sticker chart

( ) Other (Specify) ____

Collector: ________________________________ **Date of record**: _____________________

Additional information:

____

____

____
